# Mediterranean diet in axial spondyloarthritis: an observational study in an Italian monocentric cohort

**DOI:** 10.1186/s13075-021-02600-0

**Published:** 2021-08-20

**Authors:** Francesca Ometto, Augusta Ortolan, Davide Farber, Mariagrazia Lorenzin, Giulia Dellamaria, Giacomo Cozzi, Marta Favero, Romina Valentini, Andrea Doria, Roberta Ramonda

**Affiliations:** 1 Rheumatology Unit, Department of Medicine – DIMED, University of Padova, Via Giustiniani 2, 35128 Padua, Italy; 2 Dietetics and Clinical Nutrition Unit, Department of Medicine – DIMED, University of Padova, Padua, Italy; 3 Medicina Interna I^, Cà Foncello Hospital, Treviso, Italy

**Keywords:** Axial spondyloarthritis, Psoriasis, Psoriatic arthritis, Ankylosing spondylitis, Diet, Mediterranean diet, PREDIMED questionnaire, Biological treatment, Patient education

## Abstract

**Background:**

Little evidence is available about the impact of diet on disease activity of axial spondyloarthritis (axSpA). This study evaluated the impact of a 6-month nutritional advice based on the Mediterranean diet on the disease activity of axSpA.

**Methods:**

We prospectively collected the information of a group of axSpA patients who were offered nutritional advice for a 6-month period, who were compared to axSpA patients followed at the same center who were not on a specific diet. A nutritionist gave suggestions for dietary modification at baseline and thereafter every 2 months until month 6. Adherence to the Mediterranean diet was evaluated with the PREDIMED questionnaire ranging from 0 (no adherence) to 10 (optimal adherence); disease activity was evaluated with ASDAS-CRP. A multivariable regression analysis was conducted to identify independent predictors of PREDIMED and of ASDAS-CRP improvement (improvement ≥ 20% of each score).

**Results:**

A total of 161 patients were included: 81 receiving nutritional advice and 80 controls; 47 in the nutritional group and 63 controls had complete information until month 6. Overall, 40 (36.4%) were females, the mean age was 51.7 ± 1.3 years, and 58 (52.7%) were affected with psoriasis. No relevant change of anthropometric or laboratory measures was observed in either group. Adherence to the Mediterranean diet was moderate (PREDIMED score 6.7 ± 1.8 at baseline; 7.6 ± 2.1 at month 6) and improved more in the nutritional group compared to controls (*p* = 0.020). Predictors of a PREDIMED improvement ≥ 20% were receiving nutritional advice (OR 4.53, 1.36–15.1, *p* = 0.014), age (per 10-year increase OR 1.05, 1.02–1.68, *p* = 0.007), and BMI (OR 0.77, 0.63–0.9, *p* = 0.006). An ASDAS-CRP improvement ≥ 20% was more frequent in the nutritional group compared to controls (*p* = 0.020). A PREDIMED improvement ≥ 20% was associated with a ASDAS-CRP improvement ≥ 20% (OR 6.75,1.8–25.3, *p* = 0.005). Psoriasis and disease duration were negatively but not significantly associated to the ASDAS-CRP improvement.

**Conclusions:**

Improving adherence to the Mediterranean diet may have a beneficial impact on the activity of axSpA. Patients with a lower BMI and older patients are less prone to modify their diet towards the Mediterranean diet following nutritional advice. Patients with psoriasis may have a limited benefit from dietary improvement.

**Study registration:**

Protocol No. 52723, Padova Hospital Medical Ethical Committee (October 11, 2010).

**Supplementary Information:**

The online version contains supplementary material available at 10.1186/s13075-021-02600-0.

## Introduction

Axial spondyloarthritis (axSpA) is a group of debilitating, chronic, rheumatic diseases characterized by inflammation and new bone formation, mainly involving the spine and the sacroiliac joints. People living with axSpA often turn to lifestyle interventions to complement pharmacological treatment with a particular interest on diet [1]. Among environmental factors, diet has been suggested as a potential modifiable factor to improve inflammation in different rheumatic conditions [2–7]. Mediterranean diet (MD) has shown a protective role in terms of cardiovascular morbidity and overall mortality [6, 8]. MD involves high consumption of olive oil, unrefined carbohydrates, fresh and dried fruit, vegetables, and fish; reduced intake of dairy products and red meat; and moderate red wine consumption; MD is very rich in antioxidants and particularly n-3 polyunsaturated fats, alpha-linoleic acid (ALA), eicosapentaenoic acid (EPA), and docosahexaenoic acid (DHA). There is evidence that in rheumatoid arthritis, these nutrients may have a beneficial effect by reducing inflammation, reducing pain and structural damage, and possibly reducing the incidence of the disease [8–13].

There is an unmet need to understand the effects of MD on axSpA, characterized by an increased cardiovascular risk [14–16]. A considerable issue in studies concerning the MD is to assess the adherence to MD. In the PREDIMED trial, a large case-control study on cardiovascular risk, a 14-item questionnaire was introduced with this aim: PREDIMED is consistent with other dietary questionnaires to evaluate MD and is more feasible in clinical practice [17].

We conducted a prospective study in a monocentric cohort of axSpA patients who received a 6-month nutritional advice based on the MD. We evaluated the impact of the nutritional advice on dietary habits, assessed with the PREDIMED questionnaire, and on axSpA disease activity in these patients compared to controls.

## Patients and methods

AxSpA patients followed at the Padova University Hospital (Veneto Region, Italy) were offered advice by a nutritionist for a 6-month period, starting in May 2019. We collected the information of the patients in the nutritional group (N) and of a comparable group of consecutive axSpA patients who did not receive nutritional advice, controls (C). All patients were assessed every 6 months (T0 and T6) by a rheumatologist trained in the clinical assessment of axSpA and blinded to the study group. Patients in N underwent the nutritional evaluation in the day of the visits (T0 and T6) and in between every 2 months. Clinical assessment, laboratory tests, and assessment of the adherence to the MD and of cardiovascular risk were collected for all patients at T0 and T6.

### Patients

We included patients who underwent two consecutive rheumatological assessments since May 2019. Only patients fulfilling the following criteria were included in the analysis: age ≥ 18 years, diagnosis of axial involvement (axSpA) according to ASAS 2009 criteria [18], and stable (≥ 6 months) biological/targeted synthetic disease-modifying antirheumatic drug (b/tsDMARD) treatment. Exclusion criteria were rheumatological conditions other than axSpA, ongoing specific diet or optimal MD, and concomitant diabetes mellitus/metabolic syndrome. Patients were allowed to change treatment in case of suboptimal disease control. However, in order to ensure a homogenous cohort of patients with stable treatment, patients with very high disease activity (i.e., ASDAS-CRP ≥ 3.5) were excluded. Eighty-one patients in N and 80 controls were consecutively enrolled.

The local medical ethical committee approved the study (Protocol No. 52723), and informed consent was obtained from all patients at study inclusion. The study was conducted in compliance with the Declaration of Helsinki of 1975/1983.

### Nutritional advice

Patients in N underwent an interview with a nutritionist at T0. Dietary habits were assessed through the PREDIMED questionnaire [17] and through a 24-h recall of meals consumed the previous day. Based on these evaluations, the nutritionist suggested dietary modifications in order to improve their adherence to the MD. Key recommendations of the nutritionist were as follows: fractionate daily caloric intake into 3 main meals and two snacks; ensure a daily caloric intake mainly consisting of carbohydrates (55%) preferably with low glycemic index, followed by fats (20–30%) and proteins (10–15%); include adequate amounts of fibers (25–30 g daily) and water (1.5–2 l daily); consumption of vegetables, fruits, sugar-free cereals, and olive oil in every meal; use of olive oil as the main culinary fat; daily consumption of low-fat dairy products and nuts and a moderate wine consumption; weekly consumption of fish, white meat, and legumes; reduced weekly consumption of red meat, eggs, and potatoes; limit the use of salt; and only occasional consume of pastries, sweets, and soft drinks. Importantly, adoption of a non-sedentary lifestyle and regular physical activity were also recommended (Additional file 1) [18]. Every 2 months, the nutritionist asked the patients about their dietary habits and recalled the dietary modifications suggested at T0.

### Clinical assessment

At T0, sociodemographic information and information about disease history and treatments were collected. Clinical assessments were collected at T0 and T6 and included weight, height, body mass index (BMI), blood pressure, tender joint count (TJC), swollen joint count (SJC), dactylitis, Bath Ankylosing Score Metrology Index (BASMI), Maastricht Ankylosing Spondylitis Enthesitis Score (MASES), Leeds Enthesitis Index (LEI), Psoriasis Area Severity Index (PASI), physician assessment of disease on visual analogic scale (VAS), and patient-reported outcomes (PREDIMED questionnaire, the Italian version of 5-item compliance questionnaire for rheumatology (I-CQR5) [19], Bath Ankylosing Spondylitis Disease Activity Index (BASDAI), Bath Ankylosing Spondylitis Functional Index (BASFI), Health Assessment Questionnaire (HAQ), patient VAS global, and patient VAS pain). Ankylosing Spondylitis Disease Activity Score (ASDAS) with C-reactive protein (CRP) and erythrocyte sedimentation rate (ESR) scores were also computed. The following laboratory measures were collected at T0 and T6 for disease activity assessment, diet, and cardiovascular risk monitoring in clinical practice [16]: hemoglobin (HB), white blood count (WBC), platelets, (PLT) ESR, CRP, urate, glycated hemoglobin (HbA1c), fasting blood glucose (FBG), low-density lipoprotein cholesterol (LDL-c), high-density lipoprotein cholesterol (HLD-c), triglycerides, total cholesterol (TC), glutamic oxaloacetic transaminase (GOT), glutamic pyruvic transaminase (GPT), gamma-glutamyltransferase (GGT), creatinine, and thyroid-stimulating hormone (TSH).

### Dietary and lifestyle assessments

In both groups, patients were administered the Italian version of the PREDIMED questionnaire (Additional file 2) at T0 and T6. The score is computed as the sum of scores of each question, with higher values indicating a higher adherence to the MD (≤ 5 low adherence, 6–9 moderate adherence, ≥ 10 high adherence). Questions about continuous physical activity (yes/no) and the frequency of physical activity (< 1/weekly; 1/weekly; ≥ 2 weekly) were also administered.

### Cardiovascular risk assessment

Cardiovascular risk assessment in this study was evaluated with the systematic coronary risk evaluation (SCORE) and CUORE indices at T0 and T6. In chronic inflammatory arthritis, SCORE for the appropriate country (i.e., low-risk countries for Italy) is recommended [20]; alternatively, the cardiovascular risk index recommended by national guidelines may be used, such as the Italian CUORE [21]. The computation of each index is detailed in Additional file 3.

### Treatments

b/tsDMARDs considered were adalimumab, certolizumab pegol, etanercept, golimumab, infliximab, secukinumab, ustekinumab, and apremilast; csDMARD were sulfasalazine and methotrexate. NSAIDs and low-dose corticosteroid treatment were also admitted.

### Study outcomes

To evaluate an improvement in the adherence to the MD, the T0–T6 change in the PREDIMED score was considered. An improvement ≥ 20% of the T0 value was considered as a positive outcome. As a measure of clinical improvement, the ASDAS-CRP index was considered, which comprehends both clinical and laboratory measures [22]. The CRP-based index was also chosen, since it is required for the medical prescription of rheumatological treatments in the Veneto Region. The T0–T6 change was computed and an improvement ≥ 20% of the T0 value was considered as a positive outcome. Despite measures for clinically relevant improvement with ASDAS-CRP are available [23], a 20% cutoff was deemed appropriate in order to include also small improvements in clinical activity after a 6-month dietary modification.

### Statistical analysis

Characteristics of the patients are presented in all the study population and according to the study group. To identify potential biases in the study, univariate analysis tests were run to identify potential differences in T0 characteristics between C and N. Multivariate analysis was run to identify determinants of a ≥ 20% improvement from T0 in the PREDIMED score and in ASDAS-CRP. Variables included in the multivariate analysis were all those achieving a *p* < 0.10 in univariate analysis. Collinearity was assessed by the variance inflation factor (VIF), adopting a cutoff of VIF = 2 as an exclusion criterion. A logistic regression model was used, with a backward elimination approach. The results of multivariate logistic regression analysis are presented as the odds ratio (OR) with the corresponding 95% confidence interval (CI). Analyses were performed using SPSS version 24.0.

## Results

Among a total of 378 axSpA evaluated patients, 222 fulfilled the inclusion criteria and were willing to participate in the study; 61 were excluded because of an ongoing specific diet, other rheumatological overlapping conditions, diabetes mellitus/metabolic syndrome, or very high disease activity. One hundred and sixty-one patients were included in the study: 81 in N and 80 in C. Of these patients, 47 in N and 63 in C completed the evaluation at T6. T6 evaluations of patients included in the study were completed in April 2020.

### Characteristics of the patients

Characteristics of all patients and patients in N and C are reported in Table 1. Among 110 patients with complete data, 40 (36.4%) were females and 70 males (63.6%): the mean age was 51.7 ± 1.3 years, 58 (52.7%) patients were affected with psoriasis, and mean disease duration was 15.3 ± 9.7 years. No significant difference emerged between subjects in the two study groups at T0.

**Table 1:** Characteristics of the patients

	All patients*	N	C
Number of patients	110	47	63
Females, *n* (%)	40 (36.4)	18 (38.3)	22 (34.9)
Age, years, mean ± SD	51.7 ± 1.3	53.0 ± 1.3	49.6 ± 1.3
Schooling			
Primary school, *n* (%)	8 (7.3)	4 (8.5)	4 (6.3)
Middle school, *n* (%)	45 (40.9)	18 (38.3)	27 (42.9)
Secondary school, *n* (%)	36 (32.7)	17 (36.2)	19 (30.2)
University, *n* (%)	21 (19.1)	8 (17)	13 (20.6)
Social status			
Living with parents and family, *n* (%)	15 (13.6)	6 (12.8)	9 (14.3)
Living alone, *n* (%)	16 (14.5)	7 (14.9)	9 (14.3)
Living with partner and family, *n* (%)	71 (64.5)	29 (61.7)	42 (66.7)
Others, *n* (%)	8 (7.3)	5 (10.6)	3 (4.8)
Full- or part-time employed, *n* (%)	76 (69.1)	29 (61.7)	47 (74.6)
Smoking, current, *n* (%)	17 (15.5)	5 (10.6)	12 (19)
HLA-B27 positivity, *n* (%)	58 (52.7)	22 (46.8)	36 (57.1)
Psoriasis, *n* (%)	58 (50.7)	26 (55.3)	32 (50.8)
Uveitis, *n* (%)	7 (6.4)	5 (10.6)	2 (3.2)
Disease duration (years), mean ± SD	15.3 ± 9.7	15.7 ± 10	15 ± 9.5
Mechanism of action of current b/tsDMARD			
TNF inhibitors, *n* (%)	85 (77.3)	40 (85.1)	45 (71.4)
IL-12/23 inhibitors, *n* (%)	9 (8.2)	1 (2.1)	8 (12.7)
IL-17 inhibitors, *n* (%)	14 (12.7)	6 (12.8)	8 (12.7)
PDE4 inhibitors, *n* (%)	2 (1.8)	0 (0)	2 (3.2)
Duration of b/tsDMARD treatment overall, years, mean ± SD	5 ± 4.1	5.8 ± 4.5	4.5 ± 3.8
Low-dose b/tsDMARD treatment, *n* (%)	28 (25.5)	11 (23.4)	17 (27)
b/tsDMARD naïve patients, *n* (%)	81 (73.6)	37 (78.7)	44 (69.8)
No. of b/tsDMARD treatments, mean ± SD	1.5 ± 0.9	1.3 ± 0.7	1.6 ± 1.1
Steroid, *n* (%)	9 (8.2)	1 (2.1)	8 (12.7)
NSAID, *n* (%)	76 (69.1)	30 (63.8)	46 (73)
csDMARD, *n* (%)	14 (12.7)	5 (10.6)	9 (14.3)
Concomitant treatments, *n* (%)	62 (56.4)	27 (57.4)	35 (55.6)
Distance from clinic, km, mean ± SD	36.1 ± 47.2	31.1 ± 34.2	39.7 ± 54.9

*N* nutritional group, *C* control group, *b/tsDMARD* biological/targeted synthetic disease-modifying antirheumatic drug, *NSAID* non-steroidal anti-inflammatory drug, *csDMARD* conventional synthetic disease-modifying antirheumatic drug

*No significant differences were observed among any of the variables between the two groups at T0

### Clinical measures

Clinical and laboratory characteristics of the patients at T0 and T6 and the T0–T6 change are reported in Tables 2 and 3. No significant differences emerged between the two groups in characteristics at T0 and T6. A significant difference was observed in the ASDAS-CRP T0–T6 change which improved more in N (Δ −0.1 ± 0.7) compared to C (Δ 0.2 ± 0.8) (*p* = 0.003). A significant, but slight, worsening of the HbA1c levels was also observed in N (Δ 7.3 ± 21.5) compared to C (Δ 2.7 ± 5.6) (*p* = 0.01).

**Table 2:** Clinical measures

	T0			T6			Δ T0–T6		
	All patients	N	C	All patients	N	C	All patients	N	C
Number of patients	110	47	63	110	47	63	110	47	63
Weight, kg, mean ± SD	78.1 ± 16.6	75.9 ± 15	79.8 ± 17.6	77.9 ± 16.3	75.2 ± 14.2	79.8 ± 17.5	−0.3 ± 2.9	−0.7 ± 3	0 ± 2.8
Height, cm, mean ± SD	170 ± 10	168 ± 8	171 ± 10	–	–	–	–	–	–
BMI, kg/meters^2^, mean ± SD	26.5 ± 5.4	26.5 ± 4.3	26.6 ± 6.1	26.4 ± 5.3	26.3 ± 4	26.6 ± 6.1	−0.1 ± 0.9	−0.2 ± 0.9	0 ± 0.9
SBP, mmHg, mean ± SD	128.7 ± 14.9	128.7 ± 15	128.7 ± 15	128.9 ± 13.3	128.9 ± 13.6	128.9 ± 13.2	0.2 ± 11.9	0.2 ± 11	0.2 ± 12.7
DBP, mmHg, mean ± SD	81.1 ± 8.7	80.5 ± 8.2	81.5 ± 9.1	82.8 ± 9	81.5 ± 8.2	83.8 ± 9.6	1.7 ± 5.9	1 ± 4.8	2.3 ± 6.7
ASDAS-CRP, mean ± SD*	2.1 ± 1	2.1 ± 0.9	2.1 ± 1	2 ± 1.1	1.8 ± 0.9	2.1 ± 1.2	0.1 ± 0.8	−0.1 ± 0.7	0.2 ± 0.8
ASDAS-ESR, mean ± SD	2.2 ± 1	2.2 ± 1	2.2 ± 1	2.1 ± 1.1	1.9 ± 1	2.2 ± 1.2	0.1 ± 0.8	−0.2 ± 0.7	0.2 ± 0.8
BASDAI, mean ± SD	37.6 ± 23	37.4 ± 23.2	37.7 ± 22.9	39.3 ± 24.1	37.3 ± 23.6	41.2 ± 24.6	0.2 ± 1.8	−0.1 ± 1.9	0.4 ± 1.6
BASFI, mean ± SD	20.5 ± 21.4	21.6 ± 19.2	19.7 ± 23	19.8 ± 19.6	19.1 ± 18.8	20.5 ± 20.4	−0.1 ± 1.8	−0.3 ± 1.5	0.1 ± 2
BASMI, mean ± SD	1.6 ± 2	1.9 ± 2.2	1.4 ± 1.7	1.8 ± 2.1	1.9 ± 2.4	1.7 ± 1.9	0.2 ± 0.8	0.2 ± 0.6	0.3 ± 1
Patient VAS global, mean ± SD	35.5 ± 31.5	36.5 ± 30.8	34.8 ± 32.3	36.5 ± 29.6	34.9 ± 30	37.6 ± 29.6	1.9 ± 31.6	0.3 ± 31.8	3.1 ± 31.6
Patient VAS pain, mean ± SD	35.3 ± 29.5	35.5 ± 29.3	35.1 ± 29.9	38.4 ± 30.5	33.9 ± 30	37.5 ± 30.4	3.2 ± 27.9	−0.4 ± 26.8	3.7 ± 28.5
Physician VAS, mean ± SD	35.8 ± 28	34.7 ± 26.9	36.7 ± 28.9	32.3 ± 25.2	29.7 ± 26.2	34.3 ± 24.4	−3.5 ± 17.5	−5 ± 17.4	−2.4 ± 17.7
HAQ, mean ± SD	0.5 ± 0.5	0.5 ± 0.5	0.5 ± 0.6	0.4 ± 0.5	0.4 ± 0.4	0.5 ± 0.5	0 ± 0.3	0 ± 0.4	0 ± 0.3
TJC, mean ± SD	1.1 ± 2.3	0.8 ± 2.1	1.4 ± 2.5	0.9 ± 2	0.8 ± 2	0.9 ± 1.9	−0.3 ± 1.8	0 ± 1.6	−0.4 ± 2
SJC, mean ± SD	0.3 ± 1.3	0.4 ± 2	0.1 ± 0.5	0.2 ± 1.3	0.3 ± 1.8	0.2 ± 0.8	0 ± 1.8	−0.1 ± 2.7	0 ± 0.7
Dactylitis, *n* (%)	5 (4.5)	2 (4.3)	3 (4.8)	2 (1.8)	0 (0)	2 (3.2)	2 (1.8)	0 (0)	2 (3.2)
MASES, mean ± SD	1 ± 2.2	1.6 ± 3	0.5 ± 1.3	1.1 ± 2	1.3 ± 2.2	1 ± 1.8	0.2 ± 1.6	−0.3 ± 1.4	0.5 ± 1.6
LEI, mean ± SD	0.2 ± 0.8	0.3 ± 1	0.2 ± 0.6	0.3 ± 1	0.5 ± 1.3	0.2 ± 0.6	0.1 ± 0.9	0.1 ± 1.1	0 ± 0.6
PASI, mean ± SD	0.9 ± 1.9	1 ± 2.7	0.7 ± 1.2	0.8 ± 2.1	1.1 ± 3	0.7 ± 1.4	−0.1 ± 1.1	0 ± 0.6	−0.1 ± 1.3
I-CQR5 poorly adherent, *n* (%)	9 (8.2)	3 (6.4)	6 (9.5)	–	–	–	–	–	–
Regular physical activity, *n* (%)*	47 (42.7)	21 (44.7)	26 (41.3)	58 (52.7)	29 (61.5)	29 (46.2)	11 (10.0)	8 (17)	3 (4.8)

No significant difference was observed among any of the variables between the two groups at T0 and at T6, except for regular physical activity at T6 (*p* < 0.01)

*N* nutritional group, *C* control group, *BMI* body mass index, *SBP* systolic blood pressure, *DBP* diastolic blood pressure, *ASDAS-CRP* Ankylosing Spondylitis Disease Activity Score with C-reactive protein, *ASDAS-ESR* Ankylosing Spondylitis Disease Activity Score with erythrocyte sedimentation rate, *BASDAI* Bath Ankylosing Spondylitis Disease Activity Index, *BASFI* Bath Ankylosing Spondylitis Functional Index, *BASMI* Bath Ankylosing Score Metrology Index, *VAS* visual analogue scale, *HAQ* Health Assessment Questionnaire, *TJC* tender joint count, *SJC* swollen joint count, *MASES* Maastricht Ankylosing Spondylitis Enthesitis Score, *LEI* Leeds Enthesitis Index, *PASI* Psoriasis Area Severity Index, *I-CQR5* Italian version of 5-item Compliance Questionnaire for Rheumatology

*A significant change in ASDAS-CRP and regular physical activity was observed in the change of the two timepoints in the N compared to C (*p* = 0.003 and *p* < 0.01, respectively)

**Table 3:** Laboratory measures

	T0			T6			Δ T0–T6		
	All patients	N	C	All patients	N	C	All patients	N	C
HB, g/dl, mean ± SD	14.3 ± 1.3	14.3 ± 1.1	14.2 ± 1.4	14.4 ± 1.2	14.4 ± 1.1	14.4 ± 1.3	0.1 ± 0.9	0.1 ± 1	0.1 ± 0.8
WBC, 1/mm^3^, mean ± SD	6684.6 ± 1809.9	6681.9 ± 1722.3	6731.4 ± 1885	6911.2 ± 1750.7	6649.6 ± 1512.8	7106.3 ± 1897.3	226.5 ± 1505.4	227.7 ± 1347.8	374.9 ± 1607.5
PLT, 10^3^/mm^3^, mean ± SD	267.5 ± 229.1	241.5 ± 59.2	287 ± 298	247.5 ± 65.2	239.2 ± 60.5	253.6 ± 68.2	−20.1 ± 225.5	−12.3 ± 34.4	−33.4 ± 296.8
CRP, mg/L, mean ± SD	3.4 ± 6.2	3.2 ± 3.9	3.7 ± 7.4	3.5 ± 5.6	2.6 ± 3.1	4.1 ± 6.9	0.1 ± 6.4	−0.1 ± 4	0.3 ± 7.7
ESR, mm/h, mean ± SD	15.2 ± 17.1	13.8 ± 14.4	16.3 ± 18.9	14.1 ± 13.6	11.6 ± 10.3	16 ± 15.5	−1.1 ± 13.1	−2.2 ± 9.7	−0.3 ± 15.2
Urate, mg/dl, mean ± SD	5.5 ± 1.1	5.4 ± 1	5.6 ± 1.3	4.8 ± 1.8	4.7 ± 2	4.9 ± 1.7	−0.5 ± 1.6	−0.1 ± 0.3	−0.7 ± 1.9
HbA1c, mmol/mol, mean ± SD***	47.4 ± 15	52.5 ± 21.1	44 ± 9	59.6 ± 20.7	63.2 ± 26.1	57.7 ± 18.5	4.5 ± 13.3	7.3 ± 21.5	2.7 ± 5.6
FBG, mg/dl, mean ± SD*	105.1 ± 26.5	113.3 ± 30.4	100.9 ± 23.8	101.6 ± 27.2	98.9 ± 25	103.5 ± 28.9	2.4 ± 21.4	−7.4 ± 14.7	7.3 ± 22.9
LDL-c, mg/dl, mean ± SD	130.8 ± 36.5	132.3 ± 35	129.7 ± 38.4	130.6 ± 34.7	125.7 ± 34.8	134.4 ± 34.5	−1.2 ± 24.1	−5.2 ± 31.6	1.8 ± 16.7
HDL-c, mg/dl, mean ± SD**	54.7 ± 15.4	61.1 ± 17.5	49.8 ± 11.7	55.7 ± 17.5	59.2 ± 21.2	53 ± 13.6	0 ± 8	−1.1 ± 7.3	0.9 ± 8.5
TC, mg/dl, mean ± SD	209.7 ± 45.1	211.4 ± 41.2	208.5 ± 48.4	205.6 ± 37.2	204.9 ± 40.6	206.2 ± 34.6	2.1 ± 26	−0.1 ± 29.5	3.8 ± 23.3
Triglycerides, mg/dl, mean ± SD	123.2 ± 63.4	118.8 ± 57.3	127.4 ± 65.7	125.3 ± 57.8	115.5 ± 52.5	130.8 ± 60.6	2.5 ± 44	−3.3 ± 51.1	3.8 ± 38.1
TC/HDL-c ratio, mean ± SD	4 ± 1.4	3.8 ± 1.3	4.2 ± 1.4	4.1 ± 1.4	3.9 ± 1.3	4.3 ± 1.5	0.1 ± 0.5	0 ± 0.6	0.1 ± 0.4
GOT, U/L, mean ± SD	22.4 ± 8.7	23.1 ± 9.1	21.8 ± 8.5	23.1 ± 8.2	23.5 ± 8.6	22.9 ± 8	0.8 ± 8	0.4 ± 7.9	1 ± 8.2
GPT, U/L, mean ± SD	24.4 ± 15	24.7 ± 15.2	24.3 ± 15	25.1 ± 13.6	25.1 ± 12.6	25.1 ± 14.4	0.7 ± 13.1	0.4 ± 11.3	0.9 ± 14.3
GGT, U/L, mean ± SD	21.8 ± 19.1	23.7 ± 20.3	20.5 ± 18.1	23.5 ± 19.7	24.6 ± 23.4	22.7 ± 16.5	1.7 ± 20.2	1 ± 16.6	2.2 ± 22.5
Creatinine, mg/dl, mean ± SD	0.9 ± 0.2	0.9 ± 0.3	0.9 ± 0.2	1 ± 1.4	1.2 ± 2.1	0.8 ± 0.2	0.1 ± 1.3	0.3 ± 2	0 ± 0.1
TSH, μU/ml, mean ± SD	3.2 ± 2	2.9 ± 0.5	3.3 ± 2	3.5 ± 2.6	3.6 ± 3.6	3.4 ± 1.9	0.3w ± 3.7	0.5 ± 6.4	0.2 ± 0.4

*N* nutritional group, *C* control group, *HB* hemoglobin, WBC white blood count, *PLT* platelets, *ESR* erythrocyte sedimentation rate, *CRP* C-reactive protein, *HbA1c* glycated hemoglobin, *FBG* fasting blood glucose, *LDL-c* low-density lipoprotein cholesterol, *HDL-c* high-density lipoprotein cholesterol, *TC* total cholesterol, *GOT* glutamic oxaloacetic transaminase, *GPT* glutamic pyruvic transaminase, *GGT* gamma-glutamyltransferase, *TSH* thyroid-stimulating hormone

*A significative difference in the FBG levels was observed at T0 comparing the N and C (*p* = 0.039)

**A significative difference in the HDL-c levels was observed at T0 comparing the N and C (*p* = 0.048)

***A significative change in HbA1c was observed at T6 in the N compared to C (*p* = 0.01)

Notably, a significant difference was observed in the regular physical activity reported by patients at T6 which was more frequent in N vs. C (*p* < 0.01) (Table 2).

### The PREDIMED questionnaire

**Fig. 1:**
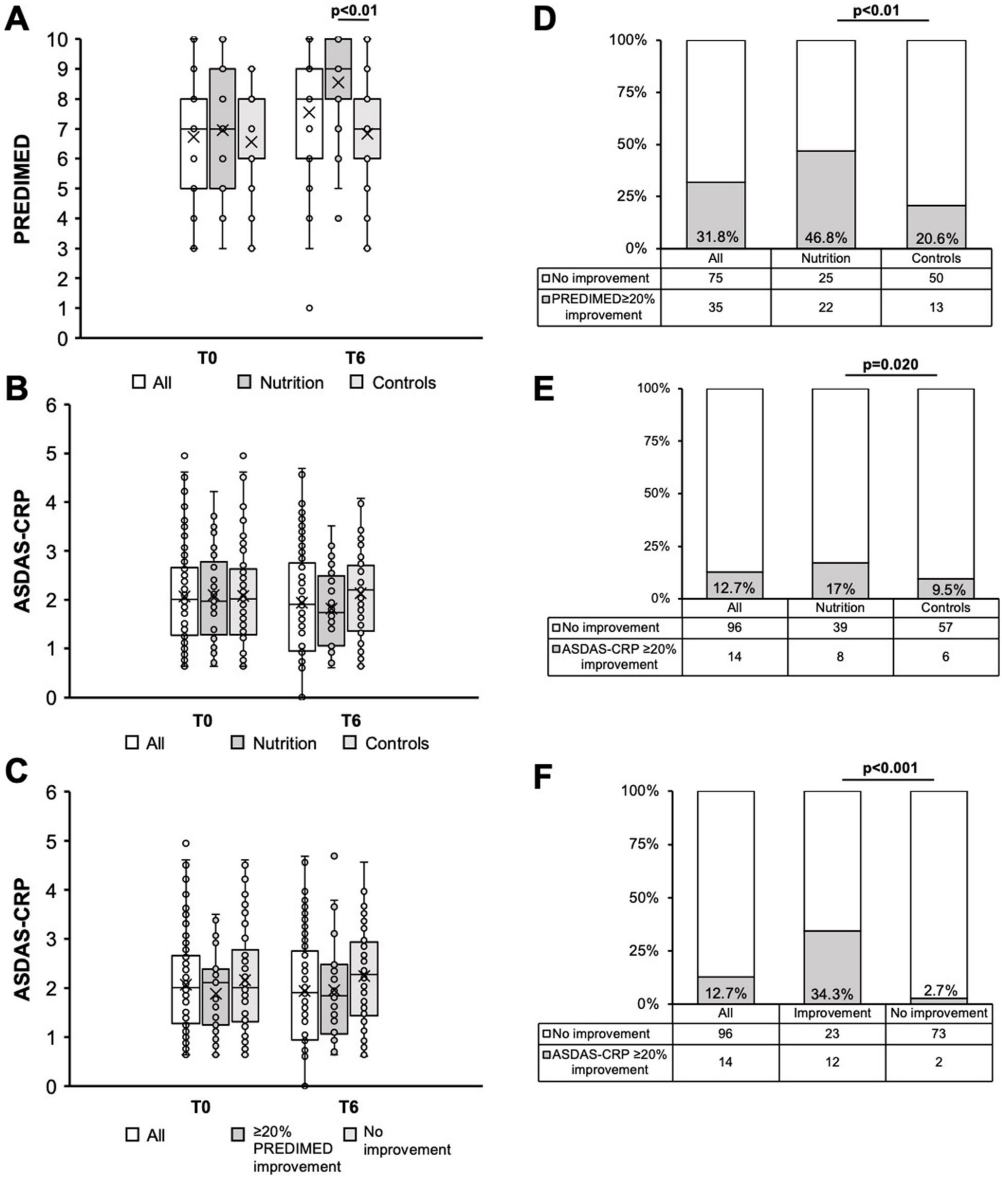
**A** PREDIMED score at T0 and T6 in all subjects, in the nutritional group, and in controls. **B** ASDAS-CRP at T0 and T6 in all subjects, in the nutritional group, and in controls. **C** ASDAS-CRP at T0 and T6 in all subjects, in subjects achieving a ≥ 20% PREDIMED improvement at T6 vs. T0, and in those who did not. **D** Frequency of ≥20% PREDIMED improvement at T6 vs T0 in all subjects, in the nutritional group, and in controls. Frequency of ≥20% ASDAS-CRP improvement in all subjects, in the nutritional group, and in controls. **F** Frequency of ≥20% ASDAS-CRP improvement at T6 vs T0 in all subjects, in subjects achieving a ≥ 20% PREDIMED improvement at T6 vs T0, and in those who did not. In box plots (**A**–**C**), mean values are represented as X

The results of the PREDIMED questionnaire at T0 and T6 and the T0–T6 change are reported in Table 4 and in Fig. 1. Overall, adherence to MD was moderate: PREDIMED score was 6.7 ± 1.8 at T0 and 7.6 ± 2.1 at T6. No significant difference in T0 values was observed in the total PREDIMED score between N (7 ± 2.1) and C (6.6 ± 1.6), while a significant difference was observed at T6 between N (8.6 ± 1.9) and C (6.8 ± 2) (*p* < 0.01) and in the T0–T6 change in N (Δ 1.6 ± 2.4) compared to C (Δ 0.4 ± 2) (*p* = 0.020). A ≥ 20% improvement in the PREDIMED from T0 was significantly more frequent in N (22/47, 46.8%) compared to C (13/63, 20.6%) (*p* < 0.01) (Fig. 1).

**Table 4:** Adherence to the Mediterranean diet assessed with the PREDIMED questionnaire

	T0			T6			T0–T6 change		
	All patients	N	C	All patients	N	C	All patients	N	C
Total score, mean ± SD*	6.7 ± 1.8	7 ± 2.1	6.6 ± 1.6	7.6 ± 2.1	8.6 ± 1.9	6.8 ± 2	1 ± 2.3	1.6 ± 2.4	0.4 ± 2
Lowest adherence, *n* (%)	25 (22.7)	10 (21.3)	15 (23.8)	16 (14)	2 (4.3)	14 (22.6)	–	–	–
Moderate adherence, *n* (%)	85 (77.3)	37 (78.7)	48 (76.2)	71 (64)	28 (59.6)	43 (67.9)	–	–	–
Highest adherence, *n* (%)	0 (0)	0 (0)	0 (0)	23 (22)	17 (36.2)	6 (9.4)	–	–	–
							**T0–T6 change**		
Question 1, *n* (%)	87 (79.1)	37 (78.7)	50 (79.4)	90 (82)	42 (89.4)	48 (75.5)	8 (7)	7 (12.8)¶	1 (1.9)¶
Question 2, *n* (%)	27 (24.5)	13 (27.7)	14 (22.2)	23 (21)	13 (27.7)	10 (15.1)	11 (9)	9 (15)¶	2 (3.8)¶
Question 3, *n* (%)	55 (50)	24 (51.1)	31 (49.2)	69 (63)	35 (72.3)	34 (54.7)	28 (25)	16 (31.9)	12 (18.9)
Question 4, *n* (%)	22 (20)	11 (23.4)	11 (17.5)	29 (26)	19 (38.3)°	10 (15.1)°	15 (14)	13 (25.5)¶	2 (3.8)¶
Question 5, *n* (%)	76 (69.1)	31 (66)	45 (71.4)	87 (79)	37 (78.7)	50 (79.2)	25 (22.7)	8 (17)	17 (26.4)
Question 6, *n* (%)	100 (90.9)	40 (85.1)	60 (95.2)	105 (94)	43 (91.5)	62 (96.2)	6(6)	5 (10.6)	1 (1.9)
Question 7, *n* (%)	78 (70.9)	34 (72.3)	44 (69.8)	93 (85)	41 (87.2)	52 (83)	21 (19)	10 (21.3)	11 (17)
Question 8, *n* (%)	17 (15.5)	7 (14.9)	10 (15.9)	18 (16)	8 (17)	10 (15.1)	8 (7)	3 (6.4)	5 (7.5)
Question 9, *n* (%)	20 (18.2)	11 (23.4)	9 (14.3)	31 (28)	17 (34)	14 (22.6)	18 (16)	8 (17)	10 (15.1)
Question 10, *n* (%)	15 (13.6)	7 (14.9)	8 (12.7)	24 (22)	17 (34)°	7 (11.3)°	16 (15)	10 (21.3)	6 (9.4)
Question 11, *n* (%)	68 (61.8)	30 (63.8)	38 (60.3)	78 (71)	34 (72.3)	44 (69.8)	25 (23)	14 (29.8)	11 (17)
Question 12, *n* (%)	23 (20.9)	14 (29.8)•	9 (14.3)•	36 (33)	26 (53.2)	10 (15.1)	19 (17.3)	14 (29.8)¶	5 (7.9)¶
Question 13, *n* (%)	73 (66.4)	35 (74.5)	38 (60.3)	80 (73)	43 (89.4)°	37 (58.5)°	20 (18)	10 (21.3)	10 (15.1)
Question 14, *n* (%)	80 (72.7)	34 (72.3)	46 (73)	88 (80)	40 (85.1)	48 (75.5)	15 (14)	8 (17)	7 (11.3)

*N* nutritional group, *C* control group

*No significant differences between C and N were observed in T0 values, while a significant difference was observed in T6 values (*p*<0.01) and in the T0–T6 change (*p* = 0.020)

•A significant difference between N and C was observed in question 12 (*p* = 0.048) at T0

°A significant difference between N and C was observed in question 4 (*p* = 0.008), in question 10 (*p* = 0.006), and in question 13 (*p* = 0.001) at T6

¶A significative difference between N and C was observed in the T0–T6 change of question 1 (*p* = 0.033), question 4 (*p* = 0.002), and question 12 (*p* = 0.004), while for question 2 it was almost significant (*p* = 0.052)

A significant difference was found at T0 in the frequency of positive response of question 12 (*mixed nuts per week*) which was higher in N. At T6, a significant difference was found in the frequency of positive responses between the two study groups in the following questions: 4 (*fruit per day*), 10 *(fish consumption per week*), and 13 (*white meat over red meat consumption*), which were higher in N. Furthermore, we observed more frequently an improvement in N compared to C in question 1 (*olive oil as the main culinary fat*), 4 (*fruit per day*), 12 (*mixed nuts per week*), and almost significant in question 2 (*olive oil consumption per day*) (Table 4 and Additional file 4).

### Predictors of PREDIMED improvement

**Fig. 2:**
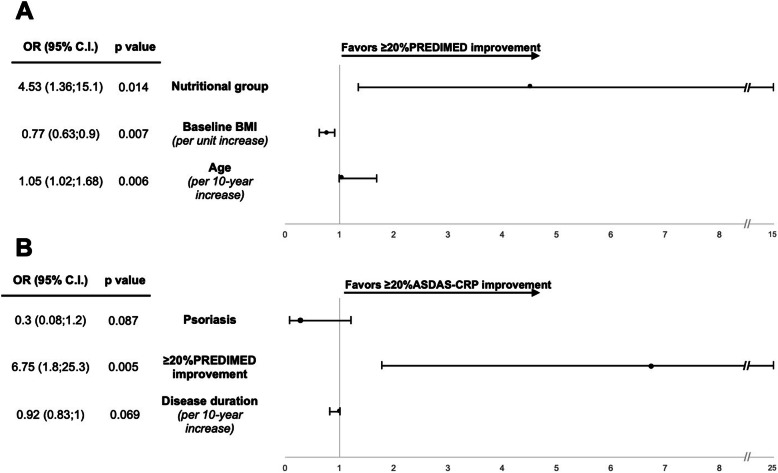
Predictors of PREDIMED and ASDAS-CRP improvement. Multivariate analysis: **A** odds for ≥20% PREDIMED total score improvement from T0; **B** odds for ≥20% ASDAS-CRP score improvement from T0. OR odds ratio; C.I. confidence interval

Characteristics of the patients and of clinical and laboratory values at T0 are reported in Additional file 5 according to the achievement of a ≥ 20% improvement in the PREDIMED score at T6. In the multivariable analysis, the following variables were included: receiving nutritional advice (group N), age, full- or part-time employment, psoriasis, disease duration, use of TNF inhibitors, concomitant treatments, BMI, patient VAS global, and CRP. No variable was excluded because of collinearity. N was associated with a higher probability of achieving an improvement of the PREDIMED score compared to C (OR 4.53, 1.36–15.1, *p* = 0.014), together with older age (per 10-year increase OR 1.05, 1.02–1.68, *p* = 0.007) (Fig. 2). BMI was associated with a lower probability of achieving an improvement of the PREDIMED score: per unit increase OR 0.77, 0.63–0.9, *p* = 0.006.

### Predictors of ASDAS-CRP improvement

Characteristics of the patients and of clinical and laboratory values at T0 are reported in Additional file 6 according to the achievement of a ≥ 20% improvement in the ASDAS-CRP score at T6. A ≥ 20% improvement in the *ASDAS-CRP* at T6 was significantly more frequent in N (8/47, 17%) compared to C (6/63, 9.5%) (*p* = 0.020) and especially in those who achieved a ≥ 20% PREDIMED improvement compared to those who did not: 12/35 (34.3%) and 2/75 (2.7%); *p* < 0.001 (Fig. 1).

In the multivariable analysis of predictors of a ≥ 20% improvement in ASDAS-CRP, the following variables were included: receiving nutritional advice (group N), ≥ 20% improvement in PREDIMED score, age, psoriasis, use of steroids, concomitant treatments, disease duration, and patient VAS global and patient VAS pain. Steroid use was excluded because of collinearity with psoriasis (VIF = 2.71). A ≥ 20% improvement in the PREDIMED score resulted in being associated with a 6-fold increased probability of achieving the ASDAS-CRP improvement (OR 6.75,1.8–25.3, *p* = 0.005). Psoriasis and a longer disease duration resulted in being negatively associated with ASDAS-CRP improvement, although non-significantly (Fig. 2)*.*

### Cardiovascular risk assessment

Overall, a low cardiovascular risk was observed in all patients in our study. At T0, SCORE was 1.7 ± 1.7 in all patients and most patients were in the lowest risk class according to CUORE (specifically 42/117, 53.7%) (Additional file 7). No significant change from T0 and in C and N were observed in SCORE or CUORE, although a trend toward a more consistent improvement of SCORE was observed in N compared to C: −0.4 ± 0.7 in N vs −0.1 ± 1 in C (*p* = 0.131). No significant difference was observed in the modification of SCORE and CUORE at T6 in the patients who improved for ≥20% in the PREDIMED score from T0.

## Discussion

This is the first study that evaluated the impact of a 6-month nutritional advice based on the MD in patients affected with axSpA. Nutritional advice was effective in improving the adherence to the MD in almost half of the patients. Older patients and those with a lower BMI were more prone to modify their diet after nutritional advice. Furthermore, patients who achieved an increase in the PREDIMED score seemed to benefit from an improvement in the disease activity of axSpA, although this may be more difficult in patients affected by psoriasis. Despite the brief study period, during which no major changes in laboratory or anthropometric measures were observed, a numerical improvement in the indices of cardiovascular risk was noted, particularly in patients receiving nutritional advice.

About one-third of the patients initially enrolled in the study dropped out and could not complete the evaluation at month 6. The main reason for lost-to-follow-up was the isolation protocol during the COVID-19 emergency in Italy in February–April 2020.

An overall moderate adherence to the MD emerged at baseline in the entire cohort with most of the patients reporting a moderate adherence to MD. This finding is consistent with a study conducted in another Italian cohort of psoriatic arthritis patients showing very similar baseline PREDIMED score and only a few patients with optimal adherence to MD [24]. After 6 months, half of the patients in the nutritional group improved their adherence to MD, and the percentage of patients showing a PREDIMED improvement ≥ 20% as compared to baseline was higher in the nutritional group than in controls. Such small changes in the PREDIMED total score were expected given that a small number of subjects are indeed reported to effectively modify their diet following a nutritional intervention over a 6–12-month period [17, 25]. Regarding specific nutrients, following nutritional advice, patients increased their olive oil, fruit, vegetables, and mixed nuts intake. Health improvements relating the specific food intake are not comparable with studies conducted in different countries, given the array of dietary habits compared to Italy [26, 27].

The multivariable analysis revealed that receiving nutritional advice increased by almost 5-fold the odds of achieving a ≥ 20% improvement in adherence to MD. The patients in the nutritional group have therefore undoubtedly benefitted from the suggestions provided by the nutritionist. Also, tight follow-up with frequent assessments may have improved the adherence to the given dietary recommendations. Patients with a lower BMI were more prone to achieve a PREDIMED improvement, which may reflect the fact that patients with an already good dietary control are more acquainted with food management and with the specific nutritional properties of the food. In addition, we observed that older patients (i.e., in their fifties) were more prone to improve their diet. This may be due to various factors: older patients may have more time to prepare meals according to the suggestion of the nutritionist, or could also be more compliant to the physician’s prescription.

No significant reduction in BMI was observed in any of the study groups, which was expected given the short duration of the study and mostly because the diet was not aimed at reducing the caloric intake. Notably, some studies in rheumatoid arthritis suggested that a slight increase in weight may also occur following MD introduction [28]. That being so, a stable BMI has not prejudiced the observed variations of disease activity in this study, which can be attributed to the diet modification [29].

A slight worsening of HbA1c was observed in the patients receiving nutritional advice. This finding may be explained by the fact that the MD may induce a higher glucidic intake compared to other diets which recommend a lower carbohydrate intake. We hypothesized that nutritional advice promoting low sugar intake (including non-sugary cereals) and complex carbohydrates with low glycemic index may be beneficial. Another possible explanation is that patients may have consumed predominantly fruits with a high glycemic index.

Adherence to MD emerged as the most significant predictor of improved disease activity in our study (≥ 20% vs. baseline, PREDIMED), resulting in a 7-fold increased likelihood of improving ASDAS-CRP as well.

Previous studies on MD nutritional interventions in rheumatic diseases have shown a beneficial effect on disease control and possibly a reduced incidence of inflammatory arthritis [2, 4, 5, 8, 9, 11, 13, 18, 22, 23, 25], though no comparable studies are currently available in axSpA patients. A recent Italian cross-sectional study, limited to psoriatic arthritis patients, confirmed the association between a better adherence to MD and improved clinical activity indices [24]. Two studies conducted in England and in Sweden showed that the implementation of MD in patients with rheumatoid arthritis was mainly associated with an improved perception of pain and disease activity but also clinical indices (e.g., CRP) [26, 27]. Likewise, in our study, both the patient’s evaluation (on a VAS scale) and the laboratory measures of inflammation (i.e., CRP) improved in the patients who initiated MD.

This improvement may be attributed to specific MD nutrients. During the study, patients receiving nutritional advice increased the consumption of olive oil and nuts which are rich in oleic acid and other n-3 polyunsaturated fats. There is evidence that n-3 polyunsaturated fatty acid supplementation—EPA and DHA, found in fish oil—associates with better disease activity scores in patients with rheumatoid arthritis [3, 11, 13]. These findings are further corroborated by studies showing that olive oil reduces inflammatory cytokine production and autoantibody development, increases T regulatory cell activation, and decreases Th17 response [7, 8, 30]. Furthermore, saturated fats and dietary fibers may also have an immunomodulatory effect on the gut microbiome in patients with autoimmune diseases [31].

Notably, although physical activity was not an outcome of the study, patients receiving nutritional advice more frequently reported increased physical activity as compared to controls. Physical activity reduces fatigue and improves sleep and innate immunity in SpA and rheumatoid arthritis patients [32, 33].

Psoriasis was negatively associated to ASDAS-CRP improvement, although this finding was not significant. Patients with psoriasis are known to have a heterogeneous disease, often representing a challenge for the rheumatologist [34]. Furthermore, these patients are often overweight, with an overlapping metabolic syndrome and a higher cardiovascular risk [14–16, 35–40]. Probably, axSpA patients with psoriasis will need studies with specific nutritional interventions.

Overall, a low cardiovascular risk was observed in all SpA patients in our cohort, irrespective of their diet, in line with previous findings of lower cardiovascular risk linked to Southern European diets [41, 42]. The lipid profile did not improve in our study nor did the blood pressure or SCORE and CUORE indices, which may be attributed to the short time of the study. Except from an improvement in blood pressure following a 6-week MD intervention in rheumatoid arthritis [26], no other study reports improvements of cardiovascular risk factors over a short time in rheumatic diseases and specifically in SpA [15].

The first limitation of this study is that the adherence to the MD was evaluated based on a questionnaire given to the patient and not with a regular meal control and hospital admission as in other studies in rheumatoid arthritis [27]. Questionnaires are prone to biased results from socially desirable answering [43]. Nonetheless, the adoption of a questionnaire allows to conduct a study in a large cohort of patients and is necessary to recall the dietary habits of the patients. Secondly, a 6-month study period may be regarded as a short time to evaluate diet and disease improvement following a dietary modification. An intrinsic problem of all studies considering nutritional intervention is to ensure an adequate and persistent adherence to the dietary modifications designed by the nutritionist: it has to be considered that a 6-month study challenges the patients’ compliance, making it difficult to maintain strict control over their eating habits for the course of study. This difficulty was one of the main reasons of drop-outs from the study, which was also affected by the isolation measures during the COVID emergency.

## Conclusions

To date, this study is the first describing the effects of the MD in patients suffering from axSpA and reporting a successful modification of dietary habits and a trend toward an improvement of disease assessment. Higher BMI and a younger age patients affected by axSpA are less prone to modify their diet towards the Mediterranean diet following nutritional advice. A better adherence to the Mediterranean diet may improve disease activity in axSpA; nonetheless, patients with psoriasis may have a limited benefit from a dietary improvement. The results may be relevant for personalized treatment approaches for axSpA patients.

## Supplementary Information

**Additional file 1.** Title: Dietary recommendations for optimal adherence to the Mediterranean Diet. Description: A table providing recommendations given by the nutritionist to the patients in the study.

**Additional file 2.** Title: The Italian version of PREDIMED questionnaire. Description: A figure with the version of the questionnaire used in the study.

**Additional file 3.** Title: Cardiovascular risk indices. Description: A table reporting the description and the computation of the cardiovascular indices for risk assessment used in the study.

**Additional file 4.** Title: PREDIMED questionnaire questions results in the nutritional group and control group. Description: A figure detailing the answers to each question of the PREDIMED questionnaire in the study.

**Additional file 5.** Title: Characteristics according to the achievement of an improvement in the PREDIMED score. Description: A table reporting the variables according to the achievement of an improvement in the PREDIMED score and the results of univariate analysis.

**Additional file 6.** Title: Characteristics according to the achievement of an improvement in ASDAS-CRP. Description: A table reporting the variables according to the achievement of an improvement in the ASDAS-CRP and the results of univariate analysis.

**Additional file 7.** Title: Cardiovascular risk indices in the study. Description: A table reporting the cardiovascular risk indices observed in the study.

## Data Availability

The datasets used and/or analyzed during the current study are available from the corresponding author on reasonable request.

## Abbreviations

ASAS: Assessment of Spondyloarthritis International Society
ASDAS-CRP: Ankylosing Spondylitis Disease Activity Score with C-reactive protein
axSpA: Axial spondyloarthritis
BASDAI: Bath Ankylosing Spondylitis Disease Activity Index
BASFI: Bath Ankylosing Spondylitis Functional Index
BASMI: Ankylosing Score Metrology Index
bDMARD: Biological disease-modifying antirheumatic drug
BMI: Body mass index
C: Control group
cDMARD: Conventional disease-modifying antirheumatic drug
CI: Confidence interval
CRP: C-reactive protein
DMARD: Disease-modifying antirheumatic drug
ESR: Erythrocyte sedimentation rate
FBG: Fasting blood glucose
GGT: Gamma-glutamyltransferase
GOT: Glutamic oxaloacetic transaminase
GPT: Glutamic pyruvic transaminase
HAQ: Health Assessment Questionnaire
HB: Hemoglobin
HbA1c: Glycated hemoglobin
HDL-c: High-density lipoprotein cholesterol
I-CQR5: Italian version of the 5-item Compliance Questionnaire for Rheumatology
LDL-c: Low-density lipoprotein cholesterol
LEI: Leeds Enthesitis Index
MASES: Maastricht Ankylosing Spondylitis Enthesitis Score
MD: Mediterranean diet
N: Nutritional group
NSAID: Non-steroidal anti-inflammatory drug
OR: Odds ratio
PASI: Psoriasis Area Severity Index
PLT: Platelets
SCORE: Systematic coronary risk evaluation
SJC: Swollen joint count
SpA: Spondyloarthritis
TC: Total cholesterol
TJC: Tender joint count
tsDMARD: Targeted synthetic disease-modifying antirheumatic drug
TSH: Thyroid-stimulating hormone
VAS: Visual analogue scale
VIF: Variance inflation factor
WBC: White blood count
