# Correction to: Mediterranean diet in axial spondyloarthritis: an observational study in an Italian monocentric cohort

**DOI:** 10.1186/s13075-021-02666-w

**Published:** 2021-11-11

**Authors:** Francesca Ometto, Augusta Ortolan, Davide Farber, Mariagrazia Lorenzin, Giulia Dellamaria, Giacomo Cozzi, Marta Favero, Romina Valentini, Andrea Doria, Roberta Ramonda

**Affiliations:** 1 Department of Medicine – DIMED, Rheumatology Unit, University of Padova, Via Giustiniani 2, 35,128, Padua, Italy; 2 Department of Medicine – DIMED, Dietetics and Clinical Nutrition Unit, University of Padova, Padua, Italy; 3 Medicina Interna I^, Cà Foncello Hospital, Treviso, Italy

**Correction to: Arthritis Res Ther 23, 219 (2021)**

**https://doi.org/10.1186/s13075-021-02600-0**

Following publication of the original article [1], the authors reported an error in the Abstract section under Conclusions.

The line “Patients with a lower BMI and older patients are ***LESS*** prone to modify their diet towards the Mediterranean diet following nutritional advice” should be corrected to the following “Patients with a lower BMI and older patients are ***MORE*** prone to modify their diet towards the Mediterranean diet following nutritional advice”.

The original article [1] has been updated.
