# Supplementary material for: Mediterranean diet in axial spondyloarthritis: an observational study in an Italian monocentric cohort
Source: Arthritis Res Ther. 2021 Aug 20;23:219. doi: 10.1186/s13075-021-02600-0 (PMC8377333; doi:10.1186/s13075-021-02600-0)
Supplement: Supplementary file 1 — Additional file 1. Title: Dietary recommendations for optimal adherence to the Mediterranean Diet. Description: A table providing recommendations given by the nutritionist to the patients in the study. [file 13075_2021_2600_MOESM1_ESM.pdf]

Additional file 1. Dietary recommendations for optimal adherence to the Mediterranean Diet.

Recommendations for optimal adherence to the Mediterranean Diet
Dietary recommendations given by the nutritionist at every assessment.

Daily caloric intake
The daily caloric intake should be provided for 55% from carbohydrates, of which 10% simple sugars; 20-30% from lipids, of which 1/3 saturated lipids, 1/3 monounsaturated, and 1/3 polyunsaturated; 10-15% from proteins, of which 1/3 of animal origin and finally 25-30g of dietary fibers are recommended.
Ration nutrients
The daily ration of nutrients must be divided into 3 main meals (breakfast lunch and dinner) and two snacks; breakfast must provide 20% of the calorie intake, lunch 35%, dinner 30% and finally each snack about 7%.
Lifestyle
It is highly recommended to adopt a non-sedentary lifestyle and to practice on a regular basis physical activity.
Specific meal recommendations
Consumption at every meal

- cereals, one or two portions per meal in the form of bread, pasta, rice or others preferably of whole wheat;
- vegetables, two or more portions per meal;
- fruit, one or two portions per meal, preferably in variety.

Daily consumption

- moderate quantities of dairy products with preference for low-fat ones;
- replace fats for cooking with extra virgin olive oil;
- consume mixed nuts and seeds;
- adequate intake of 1.5-2L of water per day;
- moderate use of wine during meals is allowed (one glass for women and two for men)

Weekly consumption

- fish and sea food, two portions or more
- white meat, two portions or more
- red meat, less than one serving per week
- eggs, two to four portions;
- legumes, more than two portions a week
- potatoes, three or less portions per week

Occasional consumption

- sweets and sugary drinks
