# Supplementary material for: Mediterranean diet in axial spondyloarthritis: an observational study in an Italian monocentric cohort
Source: Arthritis Res Ther. 2021 Aug 20;23:219. doi: 10.1186/s13075-021-02600-0 (PMC8377333; doi:10.1186/s13075-021-02600-0)
Supplement: Supplementary file 2 — Additional file 2. Title: The Italian version of PREDIMED questionnaire. Description: A figure with the version of the questionnaire used in the study. [file 13075_2021_2600_MOESM2_ESM.pdf]

Additional file 2. The Italian version of PREDIMED questionnaire

QUESTIONARIO DI VALUTAZIONE DELL'ADERENZA AL MODELLO DI ALIMENTAZIONE MEDITERRANEA

1	Utilizza l'olio di oliva come principale grasso per cucinare?	□ Sì<br>□ No
2	Quanto olio consuma al giorno (inclusi fritture, insalate, pasti fuori casa, ecc)?	□ meno di 4 cucchiai da tavola (=10 g)<br>□ ≥ 4 cucchiai
3	Quante porzioni di verdura consuma al giorno (1 porzione=200 g; considerare il contorno come una mezza porzione) ?	□ meno di 2 porzioni<br>□ ≥ 2 oppure ≥1 porzione di verdura cruda o insalata
4	Quante porzioni di frutta consuma al giorno, inclusi i succhi di frutta naturali (1 porzione=200 g) ?	□ meno di 3<br>□ ≥ 3
5	Quante porzioni di carne rossa, insaccati consuma al giorno (1 porzione=100-150 g) ?	□ meno di 1<br>□ ≥ 1
6	Quante porzioni di burro, margarina e creme consuma al giorno (1 porzione=12 g) ?	□ meno di 1<br>□ ≥ 1
7	Quanti bevande zuccherate o gassate consuma al giorno ?	□ meno di 1<br>□ ≥ 1
8	Quanto vino consuma alla settimana ?	□ meno di 7 bicchieri<br>□ ≥ 7 bicchieri (125 ml)
9	Quante porzioni di legumi consuma alla settimana (1 porzione=150 g) ?	□ meno di 3<br>□ ≥ 3
10	Quante porzioni di pesce e frutti di mare consuma alla settimana (1 porzione = 100-150 g di pesce oppure 200 g di frutti di mare oppure 4-5 pezzi)	□ meno di 3<br>□ ≥ 3
11	Quante volte alla settimana consuma dolci confezionati ?	□ meno di 3<br>□ ≥ 3
12	Quante porzioni di frutta secca (incluse le arachidi) consuma alla settimana (1 porzione=30 g)	□ meno di 3<br>□ ≥ 3
13	Consuma preferenzialmente carne di pollo, tacchino e coniglio invece che vitello, maiale, hamburgers o salsicce?	□ Sì<br>□ No
14	Quante volte alla settimana consuma vegetali, pasta, riso, altri cereali conditi con sugo semplice di pomodoro (pomodoro, cipolla, porro, olio, aglio) ?	□ meno di 2<br>□ ≥ 2
