# Supplementary material for: Mediterranean diet in axial spondyloarthritis: an observational study in an Italian monocentric cohort
Source: Arthritis Res Ther. 2021 Aug 20;23:219. doi: 10.1186/s13075-021-02600-0 (PMC8377333; doi:10.1186/s13075-021-02600-0)
Supplement: Supplementary file 3 — Additional file 3. Title: Cardiovascular risk indices. Description: A table reporting the description and the computation of the cardiovascular indices for risk assessment used in the study. [file 13075_2021_2600_MOESM3_ESM.pdf]

Additional file 3. Cardiovascular risk indices.

**SCORE[1] (for low cardiovascular risk countries)**	**CUORE [2]**
**Subjects**: men and women with no previous cardiovascular event, smokers and non-smokers, aged between 40 and 65 years, with systolic blood pressure between 120 and 180 mmHg and with a total cholesterol between 150 to 310 mg/dl.<br>**Parameters:** sex, age, systolic blood pressure and total cholesterol/high density lipoprotein cholesterol ratio.<br>**Interpretation:** the number in the cells of the chart represents the 10-year risk of fatal cardiovascular events expressed as an absolute value (%, 0-47%). No adjustment of the cardiovascular risk score computed with SCORE is recommended in axial spondyloarthritis[3].	**Subjects**: men and women who have not had a previous cardiovascular event, diabetics and non-diabetics, smokers and non-smokers, aged between 40 and 69 years, with systolic blood pressure between 90 and 200 mmHg, with total cholesterol between 130 to 320 mg/dl.<br>**Parameters:** sex, age, diagnosis of diabetes, SBP and total cholesterol .<br>**Interpretation:** the number in cells of the chart represents the 10 year risk of fatal and non-fatal cardiovascular events expressed as risk classes (%).

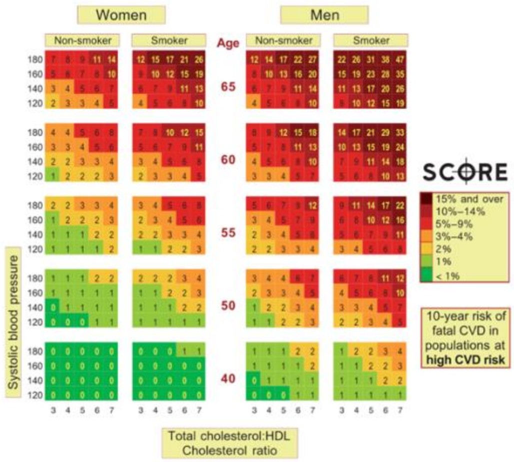

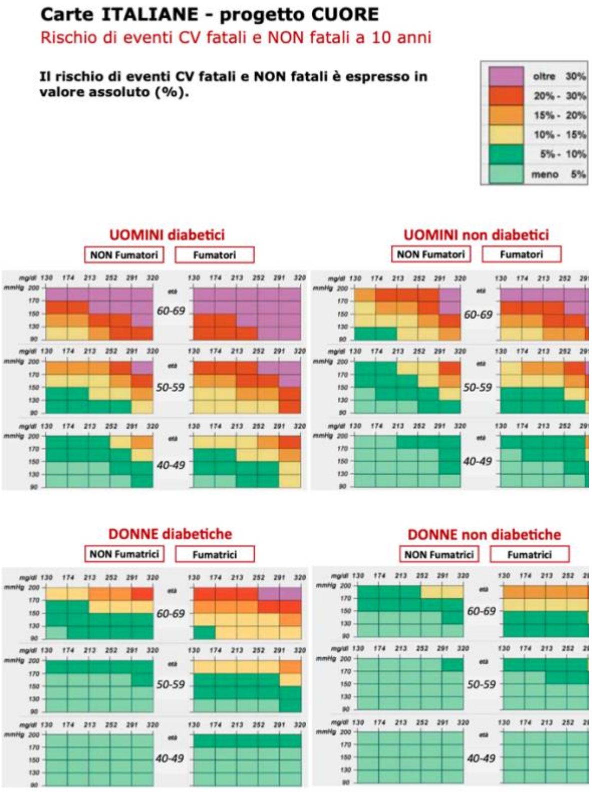

1. de Campos, O. A. M. et al. Assessment of cardiovascular risk in patients with rheumatoid arthritis using the SCORE risk index. Rev. Bras. Reumatol. ***56****, 138–144 (2016).*

2. Giampaoli, S. et al. [Assessment of the absolute global cardiovascular risk: comparison between the risk chart and the individual score of the CUORE Project]. G. Ital. Cardiol. (Rome). ***7****, 359–364 (2006).*

3. Peters, M. J. L. et al. EULAR evidence-based recommendations for cardiovascular risk management in patients with rheumatoid arthritis and other forms of inflammatory arthritis. Ann. Rheum. Dis. ***69****, 325–331 (2010).*
