# Supplementary material for: Mediterranean diet in axial spondyloarthritis: an observational study in an Italian monocentric cohort
Source: Arthritis Res Ther. 2021 Aug 20;23:219. doi: 10.1186/s13075-021-02600-0 (PMC8377333; doi:10.1186/s13075-021-02600-0)
Supplement: Supplementary file 4 — Additional file 4. Title: PREDIMED questionnaire questions results in the nutritional group and control group. Description: A figure detailing the answers to each question of the PREDIMED questionnaire in the study. [file 13075_2021_2600_MOESM4_ESM.pdf]

Additional file 4. PREDIMED questionnaire questions results in the nutritional group and control group.

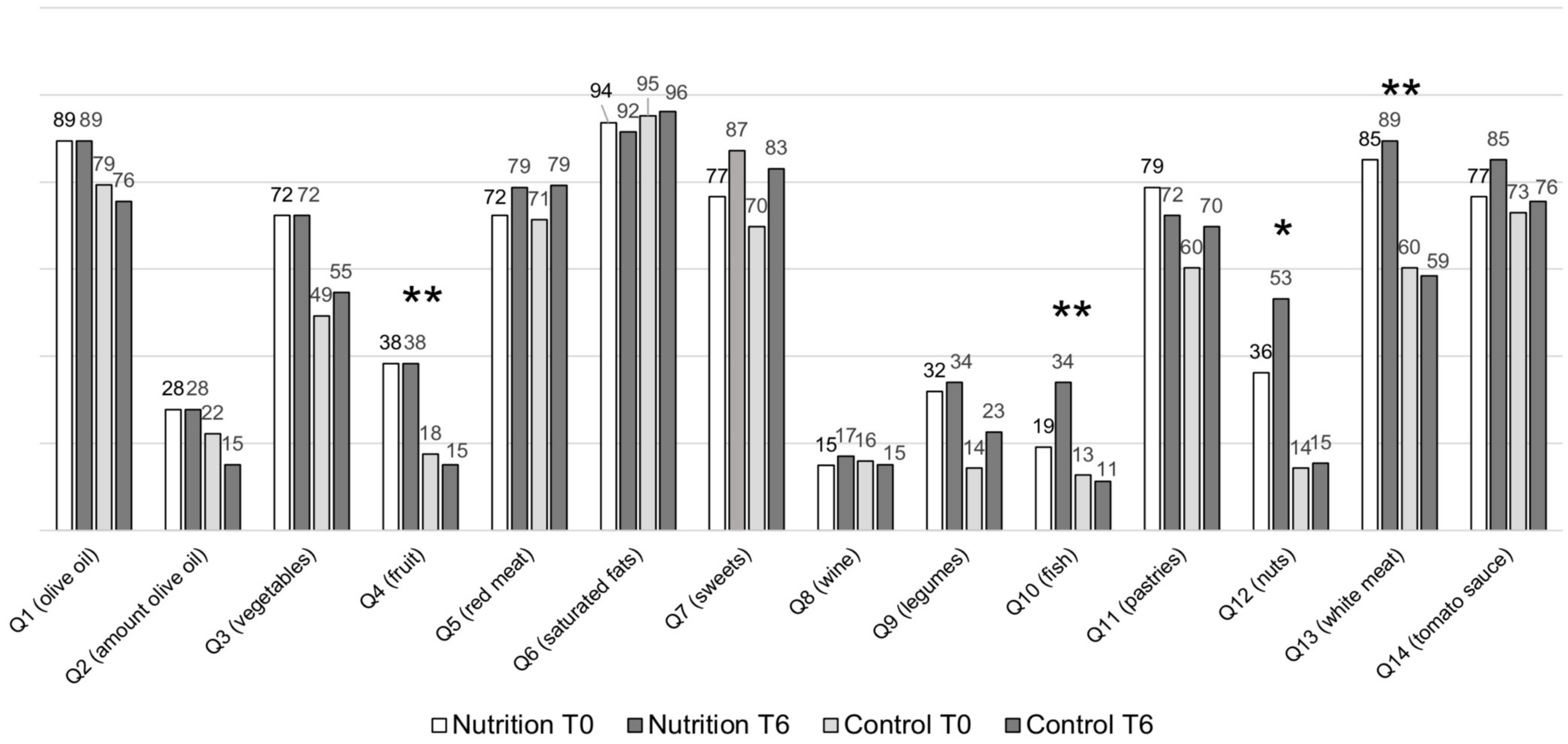

N nutritional group; *C* control group. Data at T0 and T6. Percentages of answers with score=1 (i.e. good adherence to Mediterranean diet) for each item are reported. Significant differences ($p<0.05$) between N and C are indicated as * at T0; ** at T6.
