# Supplementary material for: Mediterranean diet in axial spondyloarthritis: an observational study in an Italian monocentric cohort
Source: Arthritis Res Ther. 2021 Aug 20;23:219. doi: 10.1186/s13075-021-02600-0 (PMC8377333; doi:10.1186/s13075-021-02600-0)
Supplement: Supplementary file 5 — Additional file 5. Title: Characteristics according to the achievement of an improvement in the PREDIMED score. Description: A table reporting the variables according to the achievement of an improvement in the PREDIMED score and the results of univariate analysis. [file 13075_2021_2600_MOESM5_ESM.pdf]

Additional file 5. Characteristics of the patients according to the achievement of an improvement in the PREDIMED score

	**All**	**≥20% PREDIMED improvement**	**<20% PREDIMED improvement**
Number of patients	110	35	75
Nutritional group, n (%)¶	47 (42.7)	22 (62.9)	25 (33.3)
Females, n (%)	40 (36.4)	12 (32.9)	28 (34.6)
Age, years, mean±SD*¶	51.7±1.3	54.2±1.4	47.1±1.4
Schooling			
Primary school, n (%)	8 (7.3)	3 (8.6)	5 (6.2)
Middle school, n (%)	45 (40.9)	10 (28.6)	35 (43.1)
Secondary school, n (%)	36 (32.7)	12 (34.3)	24 (35.4)
University, n (%)	21 (19.1)	10 (28.6)	11 (15.4)
Social status			
Living with parents and family, n (%)	15 (13.6)	3 (8.6)	12 (16.9)
Living alone, n (%)	16 (14.5)	3 (8.6)	13 (16.9)
Living with partner and family, n (%)	71 (64.5)	27 (77.1)	44 (58.7)
Other, n (%)	8 (7.3)	2 (5.7)	6 (6.2)
Full- or Part-time employed n(%)*¶	76 (69.1)	27 (77.1)	49 (65.3)
Smoking, current, n (%)	17 (15.5)	5 (14.3)	12 (13.8)
Psoriasis, n (%)¶	58 (52.7)	18 (51.4)	40 (53.8)
Disease duration (years), mean±SD*¶	15.3±9.7	11.9±9.3	16.7±9.6
Mechanism of action of current b/tsDMARD			
TNFα inhibitors, n (%)¶	85 (77.3)	29 (82.9)	56 (74.7)
IL-12/23 inhibitors, n (%)	9 (8.2)	2 (5.7)	7 (7.7)
IL-17 inhibitors, n (%)	14 (12.7)	4 (11.4)	10 (13.8)
PDE4 inhibitors, n (%)	2 (1.8)	0 (0)	2 (3.1)
Duration of b/tsDMARD treatment overall, years, mean±SD	5±4.1	5.8±4.5	4.6±3.8
Low dose b/tsDMARD treatment, n (%)	28 (25.5)	8 (22.9)	20 (27.7)
b/tsDMARD naïve patients, n (%)	81 (73.6)	23 (65.7)	58 (77.3)
No. of b/tsDMARD treatments, mean±SD	1.5±0.9	1.5±0.9	1.5±1
Steroid, n (%)	9 (8.2)	1 (2.9)	8 (9.2)
NSAID, n (%)	76 (69.1)	25 (71.4)	51 (69.2)
csDMARD, n (%)	14 (12.7)	4 (11.4)	10 (13.3)
Concomitant treatments, n (%)¶	72 (65.5)	16 (45.7)	56 (74.7)
Distance from clinic, km, mean±SD	36.1±47.2	39.8±67.5	35.2±35.7
BMI, Kg/m^2, mean±SD*¶	26.5±5.4	24.7±4	27±6.3
SBP, mmHg, mean±SD	128.7±14.9	130±12.7	127±16.1
ASDAS-CRP, mean±SD	2.1±1	2.0±0.8	2.2±1
ASDAS-ESR, mean±SD	2.2±1	2±0.9	2.3±1.1

Continued on next page

Continued from previous page

BASDAI, mean±SD	37.6±23	37±24	38±22
BASFI, mean±SD	20.5±21.4	16±17	23±22
BASMI, mean±SD	1.6±2	1.8±1.7	1.7±2.1
Patient VAS global, mean±SD¶	35.5±31.5	31.3±29.2	37.7±32.1
Patient VAS pain, mean±SD	35.3±29.5	30.6±26.1	38.5±30.7
Physician VAS, mean±SD	35.8±28	30.3±23.2	38.6±28.8
HAQ, mean±SD	0.5±0.5	0.3±0.5	0.5±0.6
TJC, mean±SD	1.1±2.3	0.9±1.7	1.3±2.7
SJC, mean±SD	0.3±1.3	0.5±2.2	0.2±0.6
Dactylitis, n (%)	5 (4.5)	2 (5.7)	3 (4)
MASES, mean±SD	1±2.2	1±2.3	1±2.2
LEI, mean±SD	0.2±0.8	0.2±0.5	0.3±1
PASI, mean±SD	0.9±1.9	1.1±3	0.7±1.3
I-CQR5 poorly Adherent, n (%)	9 (8.2)	6 (17.1)	3 (4)
Physical activity, n (%)	47 (42.7)	22 (52.9)	25 (33.3)
HB, g/dl, mean ±SD	14.3±1.3	14.4±1.1	14.2±1.4
WBC, 1/mm^3, mean ±SD	6684.6±1809.9	6333.7±1366.4	6758±1919.7
PLT, 10^3/mm^3, mean ±SD	267.5±229.1	306.2±393.7	250.3±72
CRP, mg/L, mean ±SD*¶	3.4±6.2	1.9±2.1	4±7
ESR, mm/h, mean ±SD	15.2±17.1	11.8±11.8	17.8±19.8
Urate, mg/dl, mean ±SD	5.5±1.1	5.4±0.7	5.6±1.3
HbA1c, mmol/mol, mean ±SD	47.4±15	55±14.1	45.1±18.2
FBG, mg/dl, mean ±SD	105.1±26.5	115.1±33.5	103±25.9
LDL-c, mg/dl, mean ±SD	130.8±36.5	132.8±30	127.5±37
HDL-c, mg/dl, mean ±SD	54.7±15.4	58.9±18	53.8±15
TC, mg/dl, mean ±SD	209.7±45.1	220.8±42.8	200.6±44.7
Triglycerides, mg/dl, mean ±SD	123.2±63.4	120.6±60.8	121.3±71
GOT, U/L, mean ±SD	22.4±8.7	21.6±8.6	22.6±8.6
GPT, U/L, mean ±SD	24.4±15	21.8±9	25.2±17.8
GGT, U/L, mean ±SD	21.8±19.1	21.1±14.3	21±18.4
Creatinine, mg/dl, mean ±SD	0.9±0.2	0.9±0.2	0.9±0
TSH, μU/ml, mean ±SD	3.2±2	2.9±0.6	3.3±2.2

*Variables achieving a p<0.05 in univariate analysis
¶variables included in the multivariate analysis to identify predictors of ≥20% improvement in the PREDIMED score as achieving a p<0.10 in univariate analysis

b/tsDMARD biological/targeted synthetic disease-modifying antirheumatic drug; NSAID nonsteroidal anti-inflammatory drug; csDMARD conventional synthetic disease modifying antirheumatic drug; BMI body mass index; SBP systolic blood pressure; ASDAS-CRP Ankylosing Spondylitis Disease Activity Score containing C-reactive protein; ASDAS-ESR Ankylosing Spondylitis Disease Activity Score containing erytrocyte sedimentation rate; BASDAI Bath Ankylosing Spondylitis Disease Activity Index; BASFI Bath Ankylosing Spondylitis Functional Index; BASMI Bath Ankylosing Score Metrology Index; VAS Visual Analogue Scale; HAQ Health Assessment Questionnaire; TJC tender joint count; SJC swollen joint count; MASES Maastricht Anlylosing Spondilities Enthesitis Score; LEI Leeds Enthesitis Index; PASI Psoriasis Area Severity Index; I-CQR5 italian version of 5-item Compliance Questionnaire for Rheumatology; HB hemoglobin; WBC white blood count; PLT platelets; ESR erythrocyte sedimentation rate; CRP C-reactive protein; HbA1c glycated hemoglobin; FBG fasting blood glucose; LDL-c low density lipoprotein cholesterol; HDL-c high density lipoprotein cholesterol; TC total cholesterol; GOT glutamic oxaloacetic transaminase; GPT glutamic pyruvic transaminase; GGT gamma-glutamyltransferase; TSH thyroid-stimulating hormone.
