# Supplementary material for: Mediterranean diet in axial spondyloarthritis: an observational study in an Italian monocentric cohort
Source: Arthritis Res Ther. 2021 Aug 20;23:219. doi: 10.1186/s13075-021-02600-0 (PMC8377333; doi:10.1186/s13075-021-02600-0)
Supplement: Supplementary file 6 — Additional file 6. Title: Characteristics according to the achievement of an improvement in ASDAS-CRP. Description: A table reporting the variables according to the achievement of an improvement in the ASDAS-CRP and the results of univariate analysis. [file 13075_2021_2600_MOESM6_ESM.pdf]

Additional file 6. Characteristics of the patients according to the achievement of an improvement in ASDAS-CRP.

	All	≥20% ASDAS-CRP improvement	<20% ASDAS-CRP improvement
Number of patients	110	14	96
Nutritional group, n (%)*¶	47 (42.7)	8 (57.1)	39 (40.6)
≥ 20% PREDIMED improvement, n (%)*¶	35 (31.8)	12 (85.7)	23 (24)
PREDIMED change, mean±SD	1±2.3	1.7±0.1	0.7±0.4
Females, n (%)	40 (36.4)	4 (28.6)	36 (38.4)
Age, years, mean±SD*¶	51.7±13.7	54.2±13.2	50.3±1.4
Schooling			
Primary school, n (%)	8 (7.3)	0 (0)	8 (8.1)
Middle school, n (%)	45 (40.9)	5 (35.7)	40 (38.4)
Secondary school, n (%)	36 (32.7)	7 (50)	29 (32.6)
University, n (%)	21 (19.1)	2 (14.3)	19 (20.9)
Social status			
Living with parents and family, n (%)	15 (13.6)	4 (28.6)	11 (11.6)
Living alone, n (%)	16 (14.5)	2 (14.3)	14 (14)
Living with partner and family, n (%)	71 (64.5)	8 (57.1)	63 (67.4)
Other, n (%)	8 (7.3)	0 (0)	8 (8)
Full- or Part-time employed n(%)	76 (69.1)	11 (78.6)	65 (67.7)
Smoking, current, n (%)	17 (15.5)	3 (21.4)	14 (12.8)
Psoriasis, n (%)*¶	58 (52.7)	3 (21.4)	55 (57.3)
Disease duration (years), mean±SD	15.3±9.7	15±9.9	15.6±9.6
Mechanism of action of current b/tsDMARD			
TNFα inhibitors, n (%)	85 (77.3)	12 (85.7)	73 (76.7)
IL-12/23 inhibitors, n (%)	9 (8.2)	2 (14.3)	7 (5.8)
IL-17 inhibitors, n (%)	14 (12.7)	0 (0)	14 (15.1)
PDE4 inhibitors, n (%)	2 (1.8)	0 (0)	2 (2.3)
Duration of b/tsDMARD treatment overall, years, mean±SD	5±4.1	5.7±5.1	4.5±3.5
Low dose b/tsDMARD treatment, n (%)	28 (25.5)	3 (21.4)	25 (26.7)
b/tsDMARD naïve patients, n (%)	81 (73.6)	9 (64.3)	72 (76.7)
No. of b/tsDMARD treatments, mean±SD	1.5±0.9	1.4±0.6	1.5±1.1
Steroid, n (%)¶	9 (8.2)	1 (7.1)	8 (7)
NSAID, n (%)	76 (69.1)	8 (57.1)	68 (72.1)
csDMARD, n (%)	14 (12.7)	2 (14.3)	12 (14)
Concomitant treatments, n (%)¶	62 (56.4)	4 (28.6)	58 (59.2)
Distance from clinic, km, mean±SD	36.1±47.2	48.8±77.6	34±35.6
BMI, Kg/m^2, mean±SD	26.5±5.4	25.6±4.3	27.1±6.4
SBP, mmHg, mean±SD	128.7±14.9	130±14	127.4±15.2
ASDAS-CRP, mean±SD	2.1±1	2±0.9	2±1
ASDAS-ESR, mean±SD	2.2±1	2.2±1.2	2.2±1

Continued on next page

Continued from previous page

BASDAI, mean±SD	37.6±23	38±22	35±23
BASFI, mean±SD	20.5±21.4	20±21	20±21
BASMI, mean±SD	1.6±2	1.2±1.3	2.1±2.2
Patient VAS global, mean±SD*¶	35.5±31.5	38.4±33.6	32.1±30.1
Patient VAS pain, mean±SD*¶	35.3±29.5	35.8±30.7	32.5±28.6
Physician VAS, mean±SD	35.8±28	32.8±24.8	36.5±28
HAQ, mean±SD	0.5±0.5	0.6±0.6	0.4±0.4
TJC, mean±SD	1.1±2.3	0.6±1.5	1.4±2.8
SJC, mean±SD	0.3±1.3	0.1±0.4	0.2±0.6
Dactylitis, n (%)	5 (4.5)	1 (7.1)	4 (4.2)
MASES, mean±SD	1±2.2	1.2±2.6	0.8±2
LEI, mean±SD	0.2±0.8	0.4±1	0.2±0.8
PASI, mean±SD	0.9±1.9	1.8±3.9	0.6±1.2
I-CQR5 poorly Adherent, n (%)	9 (8.2)	0 (0)	9 (9.3)
Physical activity, n (%)	47 (42.7)	6 (42.9)	41 (42.7)
HB, g/dl, mean ±SD	14.3±1.3	14.6±1.3	14.1±1.3
WBC, 1/mm^3, mean ±SD	6684.6±1809.9	6173.2±1349.6	6751.1±1743.9
PLT, 10^3/mm^3, mean ±SD	267.5±229.1	221.8±57.6	289.5±292.7
CRP, mg/L, mean ±SD	3.4±6.2	3.5±3.3	3.1±6.3
ESR, mm/h, mean ±SD	15.2±17.1	17.2±24.8	15.6±15.1
Urate, mg/dl, mean ±SD	5.5±1.1	6±0	5.5±1.3
HbA1c, mmol/mol, mean ±SD	47.4±15	54±14.1	44.3±19.1
FBG, mg/dl, mean ±SD	105.1±26.5	116.3±36.7	103.3±25.9
LDL-c, mg/dl, mean ±SD	130.8±36.5	125.8±39.8	130.2±31
HDL-c, mg/dl, mean ±SD	54.7±15.4	59.2±16.1	53.3±15.9
TC, mg/dl, mean ±SD	209.7±45.1	200.7±43.8	2011.7±45.3
Triglycerides, mg/dl, mean ±SD	123.2±63.4	113±60.7	127.8±73.2
GOT, U/L, mean ±SD	22.4±8.7	21.4±7.2	22.8±8.3
GPT, U/L, mean ±SD	24.4±15	24.4±10.8	24.5±17.6
GGT, U/L, mean ±SD	21.8±19.1	22±16.4	21±18.2
Creatinine, mg/dl, mean ±SD	0.9±0.2	0.9±0.2	0.9±0
TSH, μU/ml, mean ±SD	3.2±2	1.7±2.1	23.5±2.3

*Variables achieving a p<0.05 in univariate analysis

¶Variables included in the multivariate analysis to identify predictors of ≥20% improvement in ASDAS-CRP as achieving a p<0.10 in univariate analysis

b/tsDMARD biological/targeted synthetic disease-modifying antirheumatic drug; NSAID nonsteroidal anti-inflammatory drug; csDMARD conventional synthetic disease modifying antirheumatic drug; BMI body mass index; SBP systolic blood pressure; ASDAS-CRP Ankylosing Spondylitis Disease Activity Score containing C-reactive protein; ASDAS-ESR Ankylosing Spondylitis Disease Activity Score containing erytrocyte sedimentation rate; BASDAI Bath Ankylosing Spondylitis Disease Activity Index; BASFI Bath Ankylosing Spondylitis Functional Index; BASMI Bath Ankylosing Score Metrology Index; VAS Visual Analogue Scale; HAQ Health Assessment Questionnaire; TJC tender joint count; SJC swollen joint count; MASES Maastricht Anlylosing Spondilities Enthesitis Score; LEI Leeds Enthesitis Index; PASI Psoriasis Area Severity Index; I-CQR5 italian version of 5-item Compliance Questionnaire for Rheumatology; HB hemoglobin; WBC white blood count; PLT platelets; ESR erythrocyte sedimentation rate; CRP C-reactive protein; HbA1c glycated hemoglobin; FBG fasting blood glucose; LDL-c low density lipoprotein cholesterol; HDL-c high density lipoprotein cholesterol; TC total cholesterol; GOT glutamic oxaloacetic transaminase; GPT glutamic pyruvic transaminase; GGT gamma-glutamyltransferase; TSH thyroid-stimulating hormone.
