# Supplementary material for: Mediterranean diet in axial spondyloarthritis: an observational study in an Italian monocentric cohort
Source: Arthritis Res Ther. 2021 Aug 20;23:219. doi: 10.1186/s13075-021-02600-0 (PMC8377333; doi:10.1186/s13075-021-02600-0)
Supplement: Supplementary file 7 — Additional file 7. Title: Cardiovascular risk indices in the study. Description: A table reporting the cardiovascular risk indices observed in the study. [file 13075_2021_2600_MOESM7_ESM.pdf]

Additional file 7. Cardiovascular risk indices in the study.

	**All patients***	**N**	**C**
**SCORE**			
Applicable patients, n	72	24	48
T0 value, mean±SD	1.7±1.7	1.7±1.6	1.7±1.7
T6 value, mean±SD	1.5±1.6	1.4±1.5	1.6±1.7
Change, mean±SD	-0.2±0.8	-0.4±0.7	-0.1±1
SCORE improvement ≥1 unit from T0, n (%)	18 (16.4)	10 (21.3)	8 (12.7)
**CUORE**			
Applicable patients, n	79	35	44
T0			
<5% risk, n (%)	42 (53.7)	19 (53.3)	23 (54.1)
5-10% risk, n (%)	12 (14.9)	7 (20)	5 (10.8)
10-15% risk, n (%)	20 (25.4)	7 (20)	13 (29.7)
15-20% risk, n (%)	4 (4.5)	2 (6.7)	2 (2.7)
20-30% risk, n (%)	1 (1.5)	0 (0)	1 (2.7)
>30% risk, n (%)	0 (0)	0 (0)	0 (0)
T6			
<5% risk, n (%)	38 (47.8)	15 (43.3)	23 (51.4)
5-10% risk, n (%)	17 (22.2)	10 (30)	7 (16.2)
10-15% risk, n (%)	18 (22.6)	7 (20)	11 (24.3)
15-20% risk, n (%)	4 (4.5)	3 (6.7)	1 (2.7)
20-30% risk, n (%)	2 (3)	0 (0)	2 (5.4)
>30% risk, n (%)	0 (0)	0 (0)	0 (0)
CUORE improvement ≥1 risk class from T0, n (%)	2 (2.5)	1 (1.3)	1 (1.3)

*No significant differences were observed between the two study groups.

N nutritional group; *C* control group.
